# New Methods in Digital Wood Anatomy: The Use of Pixel-Contrast Densitometry with Example of Angiosperm Shrubs in Southern Siberia

**DOI:** 10.3390/biology13040223

**Published:** 2024-03-28

**Authors:** Timofey A. Khudykh, Liliana V. Belokopytova, Bao Yang, Yulia A. Kholdaenko, Elena A. Babushkina, Eugene A. Vaganov

**Affiliations:** 1 Institute of Ecology and Geography, Siberian Federal University, 660041 Krasnoyarsk, Russia; khudykta@gmail.com (T.A.K.); kropacheva_yulechka@mail.ru (Y.A.K.); babushkina70@mail.ru (E.A.B.); eavaganov@hotmail.com (E.A.V.); 2 Khakass Technical Institute, Siberian Federal University, 655017 Abakan, Russia; 3 School of Geography and Ocean Science, Nanjing University, Nanjing 210023, China; yangbao@nju.edu.cn; 4 Department of Dendroecology, V.N. Sukachev Institute of Forest, Siberian Branch of the Russian Academy of Science, 660036 Krasnoyarsk, Russia

**Keywords:** digital wood anatomy, densitometry, image analysis, porosity, angiosperms, ring-porous, diffuse-porous

## Abstract

**Simple Summary:**

A new method, pixel-contrast densitometry, that is, measurement of the wood density based on photographs of wood cross-sections, was introduced recently for conifers. We tested it for woody plants with other types of wood structure using eight species of shrubs and small trees in Southern Siberia. Two steps were proposed to create black and white images from original photos, where cell walls are black, and empty spaces are white. In these images, the proportion of empty spaces, porosity, was measured. For all species, five measurements per ring appeared to be similar, but measurements between rings formed in different years were different. A comparison of decades of measurements with examples of three species showed the presence of common inter-annual variation in wood porosity, particularly its maximum, mean, and minimum values. These easily measured characteristics register environmental variability during their formation. Directions for further research were also discussed.

**Abstract:**

This methodological study describes the adaptation of a new method in digital wood anatomy, pixel-contrast densitometry, for angiosperm species. The new method was tested on eight species of shrubs and small trees in Southern Siberia, whose wood structure varies from ring-porous to diffuse-porous, with different spatial organizations of vessels. A two-step transformation of wood cross-section photographs by smoothing and Otsu’s classification algorithm was proposed to separate images into cell wall areas and empty spaces within (lumen) and between cells. Good synchronicity between measurements within the ring allowed us to create profiles of wood porosity (proportion of empty spaces) describing the growth ring structure and capturing inter-annual differences between rings. For longer-lived species, 14–32-year series from at least ten specimens were measured. Their analysis revealed that maximum (for all wood types), mean, and minimum porosity (for diffuse-porous wood) in the ring have common external signals, mostly independent of ring width, i.e., they can be used as ecological indicators. Further research directions include a comparison of this method with other approaches in densitometry, clarification of sample processing, and the extraction of ecologically meaningful data from wood structures.

## 1. Introduction

Digital wood anatomy, as a new direction in the study of xylogenesis and the influence of environmental factors on the seasonal growth and structure of tree rings, uses new technologies intensively and develops software for an automated analysis of tree rings [1,2,3,4,5]. One of the important problems in digital wood anatomy is the development of fast, simple, accurate, and statistically adequate methods for primary measurements [1,6,7]. A recently published paper introduced a pixel-contrast (PiC) densitometry technique designed to estimate the density profile of conifer tree rings from wood samples with polished cross-sections [8]. This is a new method of emulating densitometric measurements using digital wood anatomy and image analysis, suggested as an easily automatized and more precise alternative for previously introduced blue intensity and calculations of density from anatomical measurements. The advantages of this approach are obvious and well presented by the authors of that study. We attempted to expand the PiC densitometry technique beyond conifers, to diffuse-porous and ring-porous angiosperm woody plants, using shrubs as an example. This can be important both for expanding the scope of digital wood anatomy and for involving a wide range of woody plants in the investigation of the impact of climate change on them and of their adaptation to changing environmental conditions [9,10].

PiC densitometry operates with contrasting pixels when measuring an image of a wood cross-section. Silkin P. P. et al. [8] used a “virtual” sensor, i.e., image-analysis software, as a replacement for the X-ray densitometer. The essence of the proposed technology is that a photograph of the polished cross-surface or thin cross-section consists of contrasting pixels, points of an equal area belonging either to the cell wall or to the empty space, such as the lumen. This allows us to sum up the number of dark (walls) or light (lumen) pixels in any selected area. Knowing this, it becomes possible to find both the total areas of the cell walls and lumen and the ratio of the cell wall area to the total area of the sensor. When multiplied by the cell wall density value (wood matter density), this ratio should give the mean wood density value at the scanned area. By moving the sensor and recording successive density values, a scanning profile of a specific area of wood, including the growth ring, can be obtained.

The aim of this work is to expand the PiC densitometry method to woody plants with a wood structure of growth rings differing from that of conifers, to statistically assess the quality of the measured anatomical-densitometric characteristics of growth rings, and to assess the possibility of developing their chronologies for dendroclimatic and dendroecological studies.

## 2. Materials and Methods

### 2.1. Study Area

The work was carried out in the Western Sayan Mountains, located in South Siberia, in the Sayano-Shushensky Nature Reserve, in the vicinity of the cordon “Kurgol” in the coastal zone of the Yenisei River. The climate of the study area is sharply continental, with large daily and seasonal temperature fluctuations [11]. Precipitation is distributed unevenly throughout the year: its maximum is observed in July, and its minimum occurs in January–February. As altitude increases, temperature decreases by an average of 0.6 °C per 100 m, and annual precipitation increases by approximately 100–200 mm per 100 m [12]. The soils are loamy, shallow, and rocky, with numerous hard rock outcrops.

The Western Sayan is dominated by conifer forests, including *Pinus sylvestris* L., *Pinus sibirica* Du Tour, *Larix sibirica* Ledeb., *Picea obovata* Ledeb., and *Abies sibirica* Ledeb. in various combinations. There are also some deciduous trees, mostly *Betula pendula* Roth and *Populus tremula* L. The undergrowth is formed by the abundant growth of smaller trees and shrubs such as *Prunus padus* L., *Caragana arborescens* Lam., *Caragana altaica* (Kom.) Pojark. (aka *Caragana pygmaea* (L.) DC.), *Cornus alba* L., *Alnus alnobetula* (Ehrh.) K.Koch, *Rhododendron ledebourii* Pojark. (aka *Rhododendron dauricum* L.), *Ribes nigrum* L., *Spiraea hypericifolia* L., and others. Herbaceous cover is represented by, for example, *Veronica longifolia* L., *Actaea cimicifuga* (Schipcz.) J. Compton, *Delphinium elatum* L., *Urtica cannabina* L., *Linaria vulgaris* Mill., and many others. The moss cover is mainly represented by *Hylocomium*, *Pleurozium*, *Polytrichum,* and *Cladonia* lichens.

Data on soils and vegetation are collected from the literature [12], verbal descriptions of the Sayano-Shushensky Nature Reserve employees, and the Soil-Geographic Database of the Russian Federation (https://en.soil-db.ru, accessed on 22 March 2024).

### 2.2. Sample Collection

Samples of eight species were collected in 2022 on the territory of the Sayano-Shushensky Nature Reserve: (1) *Caragana altaica*, *Spiraea hypericifolia*—52°04.566′ N 92°13.221′ E; (2) *Caragana arborescens*, *Rhododendron ledebourii*—52°04.543′ N 92°13.073′ E; (3) *Prunus padus*, *Cornus alba*, *Alnus alnobetula*, *Ribes nigrum*—52°04.529′ N 92°13.174′ E.

The samples were taken with a hand saw in the form of a transverse section of the stem, one section per plant; the height of the sawing was 30–40 cm from the ground for *Prunus padus* and 10–15 cm for all other species.

Cambial ages (the number of growth rings from the pith to the bark) of samples used in this study varied from 5 to 42 years. Single samples were considered for *Cornus alba*, 5 years; *Ribes nigrum*, 7 years; *Spiraea hypericifolia*, 18 years; *Caragana altaica*, 19 years; and *Rhododendron ledebourii*, 37 years. Multiple samples were considered for *Alnus alnobetula*, 13–25 years; *Prunus padus*, 17–32 years; and *Caragana arborescens*, 28–42 years.

To obtain images of wood anatomy, all samples were softened by boiling in water for 2–2.5 h. Thin (20 μm) cross-sections were then prepared using a MICROM HM430 sliding microtome (Thermo Fisher Scientific, Waltham, MA, USA). Sections were stained with a mixture of 1% water solutions of safranin and Astra blue pigments and then fixed in glycerol. Images of the sections were taken at 400× magnification using a BX43 biological microscope (Olympus, Tokyo, Japan) and a ProgRes Gryphax Subra digital camera (Jenoptik, Jena, Germany).

### 2.3. Cell Structure Analysis

In this study, we used the simplest and fastest method for fixing thin stained cross-sections of wood in glycerol. This method of section preparation differs significantly from that used by permanently embedding sections in Canada balsam [13]. The most significant differences are that the cell walls of cross-sections fixed in glycerol are not dehydrated and thus swell with absorbed water; whereas in the cell walls of cross-sections fixed in Canada balsam, after dehydration of alcohol solutions (e.g., 75% and 96%), water is completely removed during fixation, which means that the walls correspond to the density of dry wood matter (about 1.5–1.53 g/cm^3^ [14,15,16]). Therefore, cross-sections fixed in glycerol have a significantly lower cell wall density (about 1.0–1.4 g/cm) [17,18,19]). Heretofore, instead of direct estimation of density (the ratio of the total wall area to the total scanning area, multiplied by wood matter density), we will use an alternative unitless value of wood porosity (the ratio of the total lumen area to the total scanning area), because it is more appropriate for the analysis of photographs of wood sections fixed in glycerol [20,21].

### 2.4. Implementation of PiC Densitometry and Software

The program implementation of the proposed method developed by T. A. Khudykh within the framework of this study has features similar to the algorithm of X-ray densitometry. The program analyzes a snapshot of the cell structure, identifying areas that belong to the cell walls or lumen.

**Figure 1:**
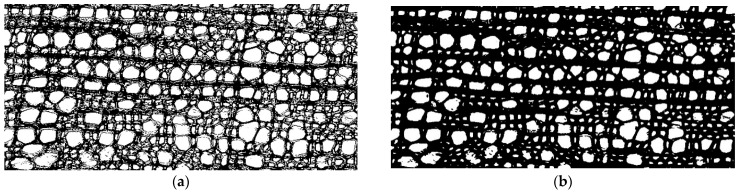
Application of the binarization algorithm using an example of the cell structure image of *Rhododendron ledebourii*: (**a**) using the original image and (**b**) after Gaussian smoothing.

Before analyzing a photograph, transformations were performed. Most binarization algorithms (classifying pixels into two groups) are extremely sensitive to noise; therefore, the Gaussian smoothing method was applied to photographs. Smoothing is often used to process photographs to improve the structure of the image and eliminate noise [22]. The Gaussian algorithm smooths out uneven color intensity values of image pixels. It is usually used to detect the contours of objects, including in histological studies [23,24]. In this study, smoothing helped to obtain clearer contours of the cell walls (Figure 1).

**Figure 2:**
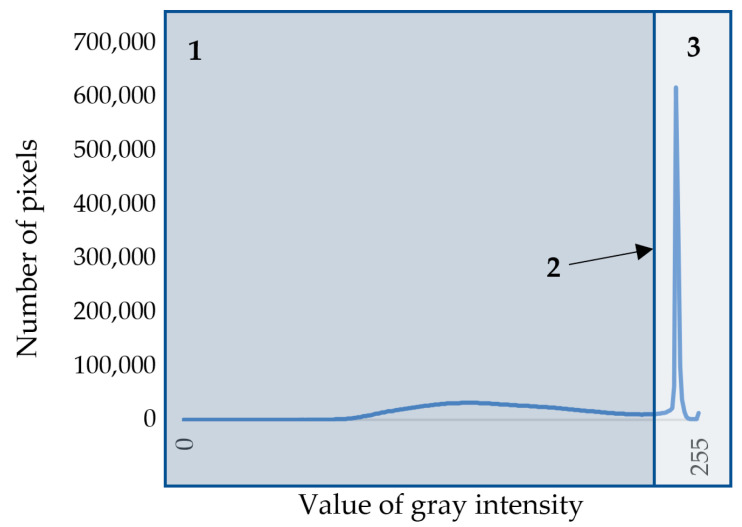
Density of the statistical distributions of pixels by shade of gray on the grayscale image of cell structure: 1—area of pixels belonging to cell walls; 2—threshold value; 3—area of pixels belonging to lumens or intercellular spaces.

Otsu’s algorithm [25] was used to separate cell walls and lumens in the image. To use this method, the image is first converted to grayscale. RGB images are characterized by three color channels (that is, each pixel in the image is characterized by three values describing the intensity of red, green, and blue colors), whereas a grayscale image is characterized by one color channel with values from 0 to 255, where 0 is black color, and 255 is white color. On the basis of the resulting grayscale image, the statistical distribution density of shades of gray in the image was calculated (Figure 2). This density is a bimodal curve, the maxima of which correspond to dark pixels of cell walls and light pixels of voids (lumens and intercellular spaces). Cell walls have to be separated from voids; for that purpose, a threshold value was calculated for each snapshot separately using Otsu’s algorithm. The essence of Otsu’s method is to set this threshold such that each class of pixel shades is as “dense” as possible [18,25]. In mathematical terms, this comes down to minimizing intra-class variance, which is defined as the weighted sum of the variances of two classes:

(1) $$\sigma_{\omega }^{2}=\omega_{1}\sigma_{1}^{2}+\omega_{2}\sigma_{2}^{2},$$

where $\omega_{1}$ and $\omega_{2}$ are shares in the distribution of the first and second classes, respectively; and $\sigma_{1}$ and $\sigma_{2}$ are standard deviations for these classes.

As a result of applying the algorithm, all “light” pixels become white, and all “dark” ones become black, converting the grayscale image into a binary one. Original photographs and binary images for all species are presented in Figure S1.

**Figure 3:**
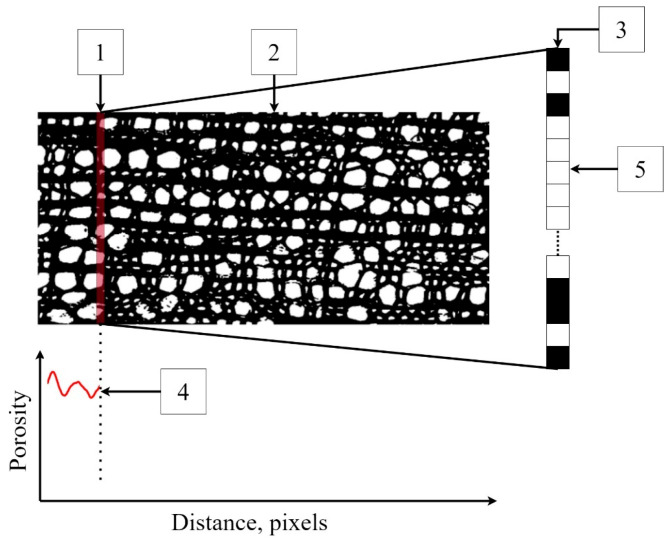
An example of the operation of a virtual sensor: 1—a 1-pixel-thick virtual sensor, 2—the scanning area, 3—the scanning line on an enlarged scale, and 4—the porosity profile (calculated as fraction of a unit). The sensor counts the number of white pixels (5) associated with the voids and calculates the porosity as its ratio to the total number of pixels on the scan line.

The program has a virtual sensor (Figure 3) in the form of a scanning line one pixel thick and an arbitrarily specified width of the scanning window. This virtual sensor moves along the length of the binary image, selecting black and white pixels at each step. The program then determines the ratio of the number of white pixels to the total number of pixels on the scanning line. After a one-pixel shift, this operation is repeated until it is completed for the entire scanning area, i.e., the growth ring.

The software created for measuring and processing wood porosity data (https://github.com/Timofey00/OpenPiCDens, accessed on 25 February 2024) was written in Python, which has a wide range of public libraries and allows for the rapid development of programs of varying degrees of complexity.

**Figure 4:**
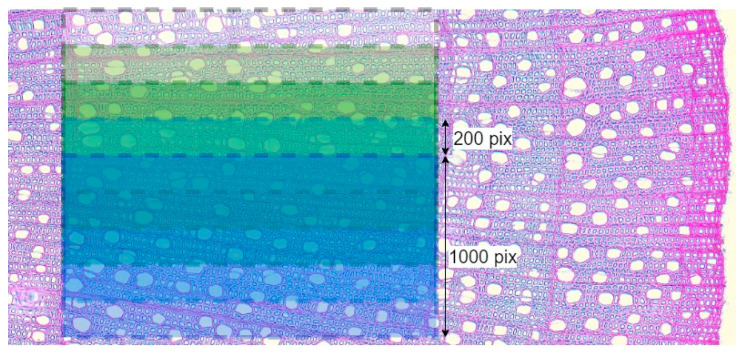
Visualization of scanning several overlapping areas on one image using the example of *Cornus alba* growth ring in 2020. For illustration, the original color image is shown here instead of the binary image with which calculations were actually performed.

A major role in the result of PiC densitometric analysis is played by the selection of the scanning area [17,18,19]. Various defects (callus, cell ruptures, etc.) can have a strong influence on the resulting quantitative ratio of void pixels to the total number of pixels (hereinafter referred to as Por), but so can rays, extremely large vessels, and other irregularities in the wood structure. To investigate and mitigate the impact of such disturbances on the resulting profile of Por, for each growth ring, Por was calculated five times. The scanning area was selected for the first iteration, with a scanning line length of 1000 points, and after each iteration, it was shifted by 200 points (Figure 4), i.e., a moving window was applied with a 1000-pixel width and a 200-pixel step. Such a window width was chosen to make the scanning area overlap several rows of large vessels in ring-vascular species, making it possible to smooth out structural inhomogeneities more effectively.

To calculate and compare Por values of different species and different weather conditions during ring development, photographs of growth rings from 2018 to 2022 for one sample of each species were used.

For the three long-living species, *Caragana arborescens*, *Prunus padus*, and *Alnus alnobetula*, long-term series of ring widths (measured from photographs) and porosity profiles were obtained with repetition of 12–13 samples, using most of each sample except first years from the pith, where the most significant nonlinearity of the growth ring boundaries was observed.

Pearson paired correlations were used to compare time series between each other. Correlation analysis included inter-series correlations [26] for five measured porosity profiles (135–1074 pixels long) within the ring and between 10 and 13 series (13–32 years long; tree-ring width, mean, maximum, and minimum value of porosity) within each of the three long-living species. Correlations between local averaged chronologies of tree-ring width, mean, maximum, and minimum porosity were also calculated, and their significance was estimated with a two-tailed *t*-test.

## 3. Results and Discussion

### 3.1. Influence of the Selection of the Scanning Area on the Measured Porosity Profile

To estimate the effect of the scanning area selection on the values of Por, the correlations of the Por profiles for each scanning area and the averaged Por profile over five measurements were calculated in a single growth ring image for each species (Figure S2 and Table 1). The correlations were quite high, e.g., the lowest correlation coefficient was observed for one of the scanning areas of *Spiraea hypericifolia* (*r* = 0.60 at *p* < 0.01), and all other values exceed *r* = 0.79. Such high correlations indicate a connection between measurements in different scanning areas and the presence of common variability between them, i.e., a common external signal.

### 3.2. Comparison of Typical Porosity Profiles of the Studied Species

Studies applying methods of image analysis on the wood structure are more often devoted specifically to conifer trees due to regular, uniform, and easy-to-quantify cell anatomy. However, this study examined porosity profiles for other types of wood: diffuse-porous (*Prunus padus*, *Cornus alba*, *Alnus alnobetula*, *Rhododendron ledebourii*, *Ribes nigrum*), semi-ring-porous (*Spiraea hypericifolia*), and ring-porous (*Caragana arborescens*, *Caragana altaica*) (Figure S3).

The porosity profile of ring-porous woody plants differs significantly from that of conifers [27,28]. The porosity profile of conifer wood is characterized by a smooth transition from earlywood to latewood; however, high-frequency fluctuations may be observed in it because of the regular arrangement of tracheids in the cell structure of conifer wood. Ring-porous wood has a much more distinctive transition from earlywood to latewood. The ring-porous structure of wood is typical of many species of deciduous trees and shrubs of the temperate zone; its earlywood, containing mainly the larger vessels, develops at the beginning of the season, whereas the rest of the ring contains fibers, parenchyma, and a few smaller vessels. Due to the disproportionate water conductivity of the largest vessels, earlywood accounts for more than 90% of the water supply by the growth ring [29]. As a result, porosity profiles for two *Caragana* species show porous earlywood at the beginning of the growth ring and then a relatively sharp transition to a porosity several times lower in latewood. In a shrub with a transitional wood structure, *Spirea*, a similar but smoother curve of the porosity profile was observed.

No less interesting are the porosity profiles of woody plants with diffuse-porous wood. For example, the porosity of *Rhododendron* wood has a clear tendency to decrease almost linearly throughout the growth ring. *Cornus*, *Alnus*, and *Prunus* woods do not show such a clear trend across the entire ring. There, porosity is reduced only in the very last cells at the border of the ring. Note that the rings of these diffuse-porous plants show high-frequency fluctuations in the arrangement of vessels, which are comparable in amplitude to porosity dynamics along the ring and are repeated for different scanning lines. Finally, the most interesting example of diffuse-porous wood is *Ribes*. Here, the high-frequency oscillations are maximally pronounced and show (especially in the later part of the ring) a very synchronous alternation of vessels and denser tissue.

All species also showed year-to-year variability in their porosity profiles (Figure S3). In ring-porous species, a small second increase in the porosity profile can be seen after a transition from more porous earlywood to less porous latewood, which is associated with the presence of clusters of small vessels. However, the presence of such a second porosity maximum in latewood is not mandatory. In *Caragana arborescens*, it is expressed in 2018 (at the beginning of the zone), 2021, and 2022 (closer to the middle of the latewood zone). In *Caragana altaica*, the most pronounced such fluctuation was in 2019, and weaker fluctuations were noticeable in 2018 and 2021. The proportion of earlywood also changed from year to year in both species, but its variability was much higher in the wood of *Caragana altaica*.

The porosity profile of *Spiraea hypericifolia*, which has semi-ring-porous wood, is smoother, although similarity with the ring-porous species has also been recorded in some years (for example, 2021, 2019). This species, similarly to both ring-porous shrubs, is characterized by smooth porosity profile curves without pronounced high-frequency fluctuations.

In diffuse-porous wood, the interannual variability of the porosity profile has different patterns. In almost all species under consideration, every year, the trend of a sharp decrease in the porosity profile at the end of the growth ring persisted, with a nearly horizontal line before that. In *Ribes nigrum*, synchrony in vessel formation was maintained annually, reflected in many pronounced short-term variations in the porosity profile throughout the ring, particularly evident in the broad rings developed from 2018 to 2020. *Ribes nigrum* was the fastest-growing shrub of all investigated; thus, the probable temporal frequency of these fluctuations, even with a very rough estimate of the duration of cambial activity (cell division) at approximately 3 months, was no more than a few days in wide rings. It can be assumed that these synchronous oscillations between the production of vessels and dense tissue, which result in rows parallel to the border of tree rings, are somehow connected with short-term inner rhythms, perhaps including circadian rhythms, the impact of which on physiological processes and the morphology of growing plant tissues through gene expression is already known [30].

In *Prunus padus* and *Alnus alnobetula*, the porosity profile usually decreased gradually throughout the entire ring. However, *Alnus* also had rings in which the porosity profile increased slightly before transition to latewood (2020, 2022). The degree of expression of high-frequency fluctuations in porosity (i.e., the regularity of vessel location and formation) varied from year to year.

The porosity profile of *Cornus alba* showed the same dynamics throughout all five years: the porosity profile decreased across the entire ring. Of the species under consideration, only *Rhododendron ledebourii* showed extremely low inter- and intra-annual variability. In all years except 2022, the porosity values were approximately 0.3 over the entire profile for this species.

In this work, we deliberately switched the object of analysis from traits of individual cells or their elements to the continuous profile of porosity. The study was aimed at a more integral assessment of wood structure and the subsequent ability to find wood characteristics responding to external conditions (including climate). For example, profiles of the porosity of ring-porous species indicate that promising indicators of structural variability may be the maximum value of porosity, the proportions of porous earlywood and dense latewood in the ring, and possibly the average porosity values in these zones. It can be assumed that the boundary between early- and latewood zones within a ring can also be determined by a certain threshold porosity value, similar to the Mork criterion (the ratio of cell wall thickness to the radial lumen diameter of 0.25 or 0.5 [31,32]) in conifer trees or to densitometric thresholds (mean or specific value of density) of earlywood-to-latewood transition [33,34,35]. A promising approach for identifying the threshold value for such a criterion could be an algorithm similar to Otsu’s method, perhaps with nonlinear transformation to make distinction easier [36,37].

For diffuse-porous wood, as our measurements show, indicators of external conditions during the season can be intra-annual trends in porosity, significant fluctuations of porosity within tree rings, as well as its minimum, maximum, and average values.

### 3.3. Comparative Analysis of Porosity Profile Indicators and Ring width Using Three Long-Living Species

**Figure 5:**
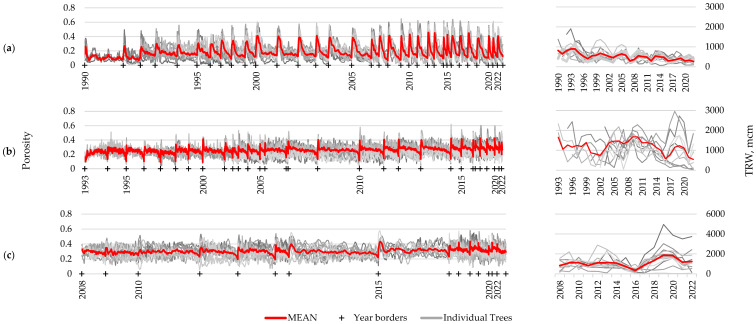
Long-term series of porosity profiles (**left**) and ring width chronologies (**right**) of *Caragana arborescens* (**a**), *Prunus padus* (**b**), and *Alnus alnobetula* (**c**). Thick red lines represent mean series, thin gray lines represent series of individual specimens; crosses and year labels mark the beginning of the labeled year. For averaging the porosity profiles between individual series, they were compressed to the shortest one within that year.

For the three longest-living species of those under consideration, long-term porosity profiles were constructed using at least 10 samples: *Caragana arborescens*—32 years, *Prunus padus*—29 years, and *Alnus alnobetula*—14 years. In addition, for these species, series of growth ring widths were measured for comparison (Figure 5). There is clearly noticeable year-to-year variation in the wood structure, expressed through porosity profiles, comparable with the differences between samples for one year. This is an indicator of the presence of certain common external drivers that determine the variability of the wood structure and synchronously influence all examined samples. Presumably, these common external drivers include climatic fluctuations. However, high-frequency intra-ring fluctuations are not synchronous between samples collected from different plants (unlike between different scanning areas within one sample), indicating their probable regulation by internal rhythms, expressed individually for each specimen. Different numbers of oscillations (vessels versus small cells) per ring among specimens may be one of the main reasons for unsynchronous high-frequency fluctuations.

**Figure 6:**
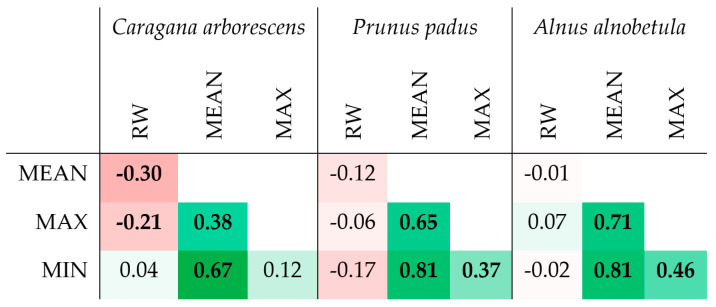
Heatmap of correlations between several porosity profile characteristics and ring width (RW) of *Caragana arborescens*, *Prunus padus*, and *Alnus alnobetula*. Bold values are significant at *p* < 0.05; all values are marked with color gradient from deep red (−1) through white (0) to deep green (+1).

In all three species, strong correlations were noted between the series of maximum, minimum, and average values of the porosity profile, which suggests functionally determined limitations and/or strict relationships between these values (Figure 6 and Figure S4). For example, all values of the porosity can be restricted by a trade-off between limited resources and hydraulic resistance of the conduits, the latter depending on species/provenance, site conditions, and distance to the stem apex [38]. At the same time, a significant relationship between the ring width and the porosity profile indicators was noticed only in *Caragana arborescens* (−0.30 and −0.21 with *p* < 0.05 for the average and maximum porosity, respectively, i.e., the wider the ring, the denser the wood).

In diffuse-porous *Alnus alnobetula* and *Prunus padus*, the mean inter-series correlation coefficients (i.e., correlations between series for individual specimens, Table 2) for maximum, minimum, and average porosity in the ring were comparable or higher than those for the ring width series, which indicates the presence of a common signal to external factors affecting growth ring formation in these indicators. Of course, for such a short series, we cannot talk seriously about the level of significance, but even these preliminary results are already indicative, since they are at least comparable with the inter-series correlation coefficients observed for other anatomical parameters of the wood structure in both conifers and deciduous trees [39,40,41,42,43]. For the ring-porous *Caragana arborescens*, despite clearly visible differences in the structure of growth rings between years, the inter-series correlation coefficient is not close to zero only for the maximum porosity. This leads us to the conclusion that for a quantitative description of the structure of ring-porous wood, other features of the porosity profile that were not investigated here may be more ecologically significant indicators of the annual dynamics of porosity.

## 4. Conclusions

PiC densitometry proved to be applicable to angiosperm woody plants with various wood structures, from ring-porous to diffuse-porous. Gaussian smoothing was proposed to minimize errors and noise, obtaining clearer contours of cell walls, and Otsu’s algorithm was used to perform classification of pixels. This allowed us to convert original color photographs of wood cross-sections into binary images, separating cell walls and empty spaces in the wood structure. The software created specifically for this purpose allows us to apply these procedures and measure porosity profiles automatically, resulting in five well-synchronized measurements and an averaged profile for each ring.

Comparison of growth rings in the same wood samples over several years demonstrated that porosity profiles register inter-annual variability of the wood structure (differences in the wood structure between rings, explained by variability of the environmental conditions and the state of the plant), as well as intra-annual variability (trends describing a sequence of earlywood and latewood, explained genetically and probably by day length as a strictly periodic factor). Testing of the more long-lived species revealed that the maximum porosity series contain external signals common among specimens of the same species; for diffuse-porous species, this is also true for mean and minimum porosity. These signals overlap among porosity variables but are mostly independent of ring width, making porosity variables more attractive as ecological indicators.

Further research can be performed in the following directions: (1) finding wood porosity or density variables most clearly registering external impacts on ring growth and development; (2) comparing PiC densitometric data with traditional densitometry and calculations of wood density based on anatomical measurements; and (3) comparing PiC densitometry performed on wood cross-sections fixed in different substances. For the sake of the first two purposes, PiC densitometric measurements should be carried out on wood samples (both angiosperms and gymnosperms) for which density and/or anatomical long-term chronologies are already developed. Resulting time series of porosity characteristics should be compared among themselves, with existing tree-ring data, and with ecological factors impacting tree growth using statistical analysis, like correlations, regression models, cluster classification, and other such methods. For the latter purpose, we suggest performing such comparisons using thin cross-sections of the same wood samples fixed in different substances.

## Figures and Tables

**Table 1:** Correlation coefficients between measurements obtained from different scanning areas and the average porosity profile. All given correlations are significant at *p* < 0.01.

Species	Scanning Area				
	1	2	3	4	5
*Cornus alba*	0.87	0.94	0.94	0.91	0.82
*Alnus alnobetula*	0.82	0.89	0.93	0.93	0.85
*Prunus padus*	0.86	0.94	0.97	0.95	0.98
*Caragana arborescens*	0.79	0.94	0.97	0.92	0.82
*Caragana altaica*	0.96	0.98	0.98	0.88	0.95
*Rhododendron ledebourii*	0.95	0.96	0.96	0.79	0.89
*Ribes nigrum*	0.91	0.95	0.92	0.91	0.79
*Spiraea hypericifolia*	0.60	0.87	0.99	0.97	0.95

**Table 2:** Mean inter-series correlation coefficients of the tree-ring parameters.

Characteristics	Species		
	*Caragana arborescens*	*Prunus padus*	*Alnus alnobetula*
RW	0.41	0.17	0.28
MEAN Por	0.09	0.53	0.17
MAX Por	0.19	0.30	0.14
MIN Por	0.03	0.47	0.29

## Data Availability

The source code of the software created for this study is available at https://github.com/Timofey00/OpenPiCDens (accessed on 25 February 2024). The raw data supporting the conclusions of this article will be made available by the authors on request.

## Supplementary Materials

The following supporting information can be downloaded at: https://www.mdpi.com/article/10.3390/biology13040223/s1, Figure S1: Photographs and binary images of the cell structure for the considered stained thin cross-sections of shrub wood; Figure S2: The process of obtaining wood porosity profiles for various species with the example of a ring developed in 2020; Figure S3: Wood porosity profiles of various species for 2018–2022; Figure S4: Dependencies of wood porosity indicators among themselves and with the growth ring width.
