# Supplementary material for: New Methods in Digital Wood Anatomy: The Use of Pixel-Contrast Densitometry with Example of Angiosperm Shrubs in Southern Siberia
Source: Biology (Basel). 2024 Mar 28;13(4):223. doi: 10.3390/biology13040223 (PMC11048334; doi:10.3390/biology13040223)
Supplement: Supplementary file 1 [file biology-13-00223-s001.zip › biology-2928648-supplementary.pdf]

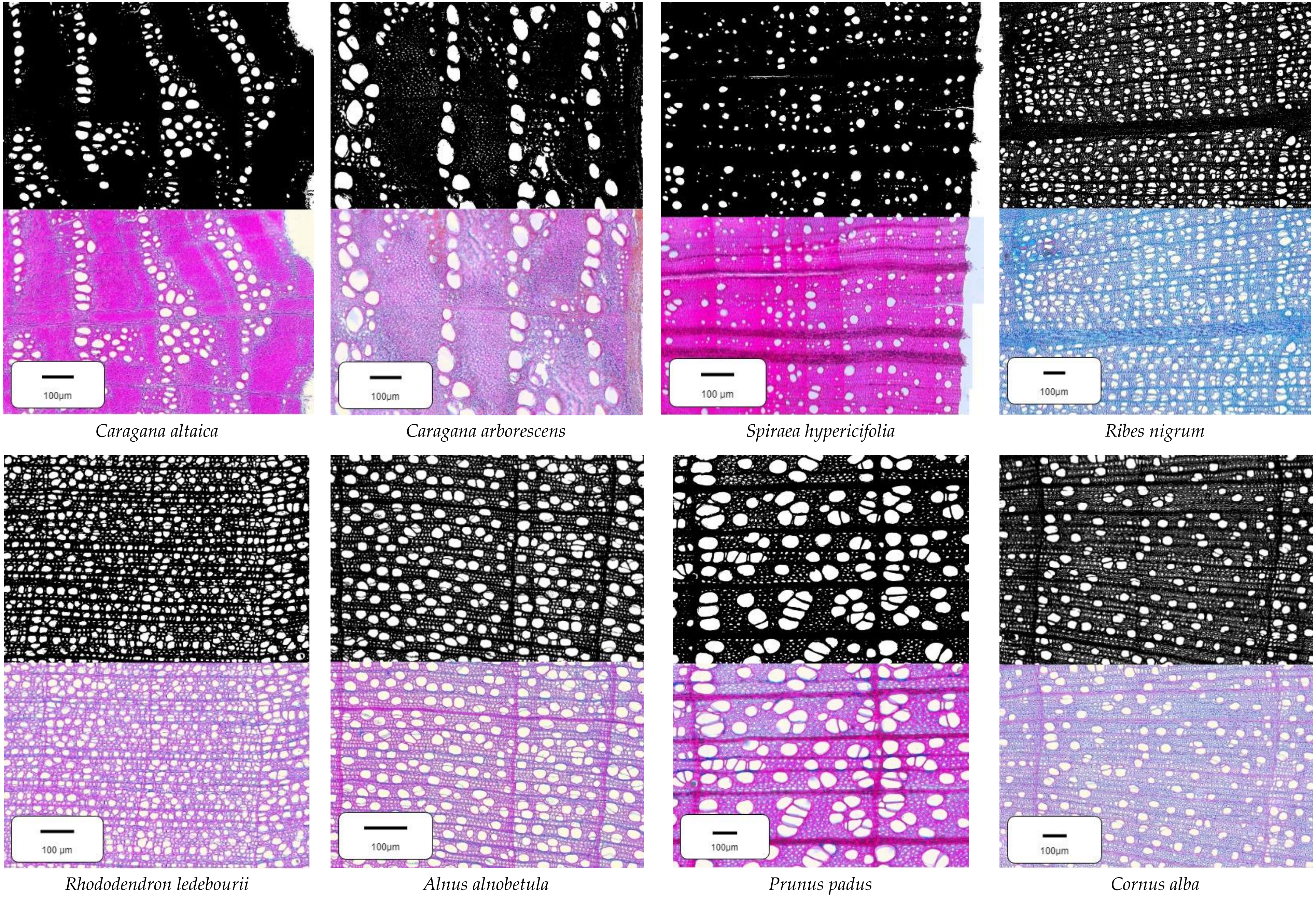

Figure S1. Photographs and binary images of the cell structure for the considered stained thin cross sections of shrub wood.

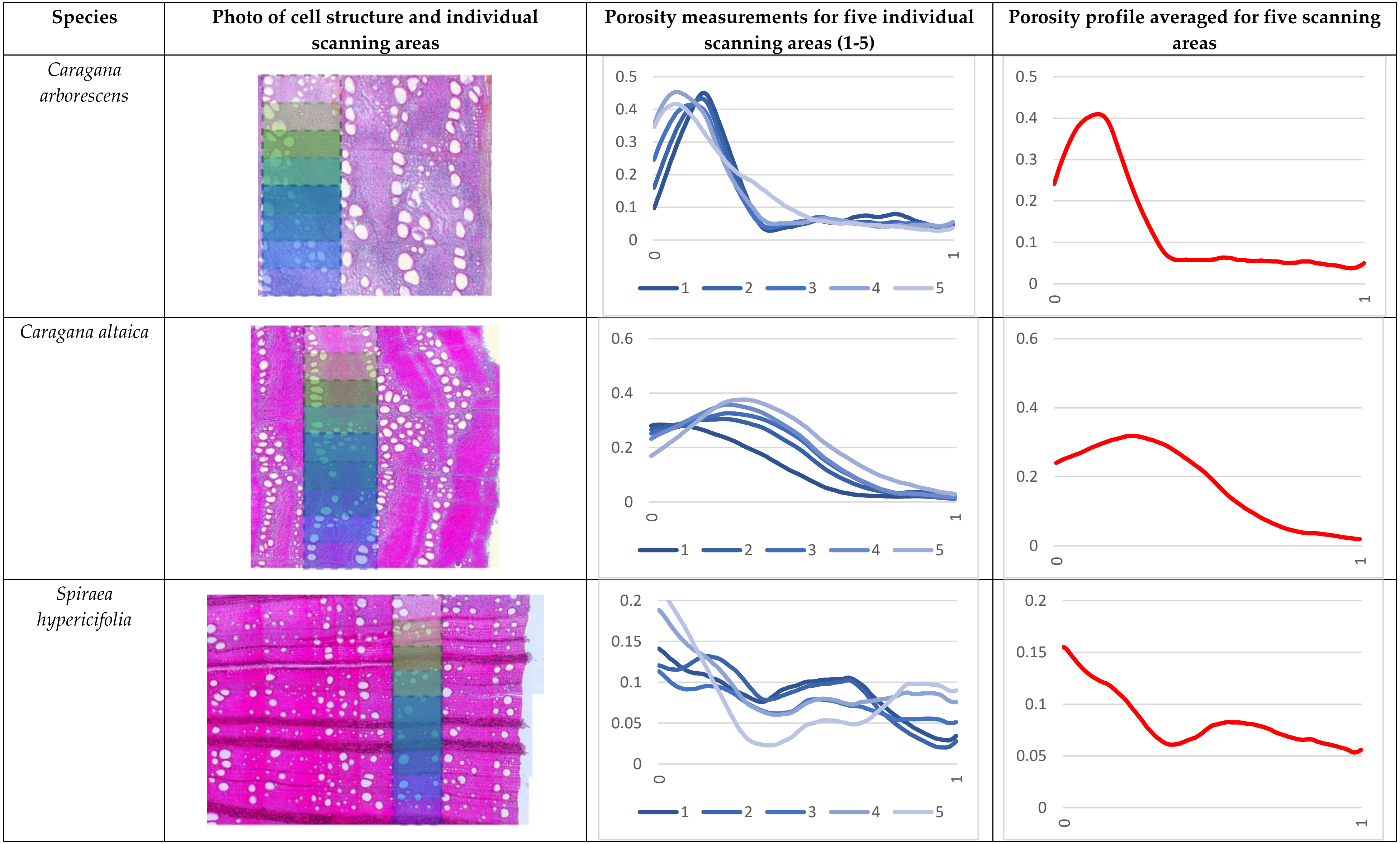

Figure S2. The process of obtaining wood porosity profiles for various species with the example of a ring developed in 2020.

Species	Photo of cell structure and individual scanning areas	Porosity measurements for five individual scanning areas (1-5)	Porosity profile averaged for five scanning areas
Cornus alba	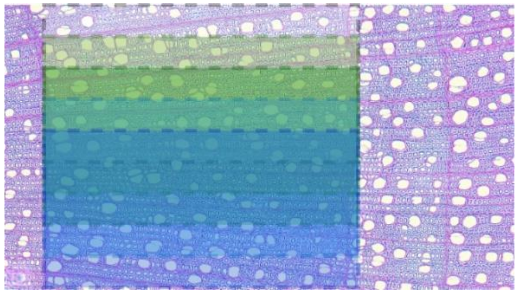	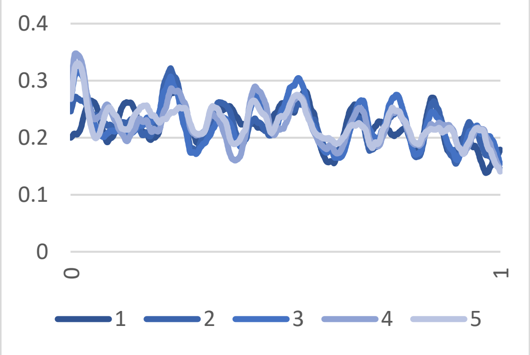	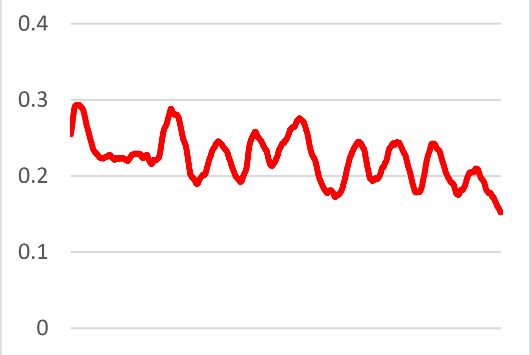
Alnus alnobetula	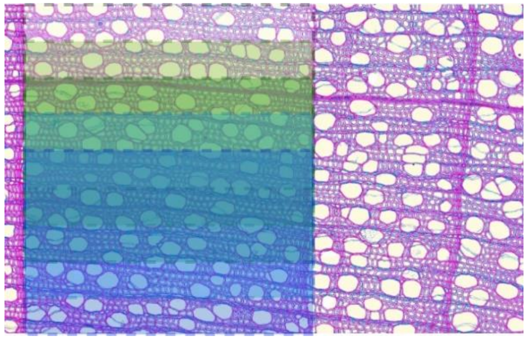	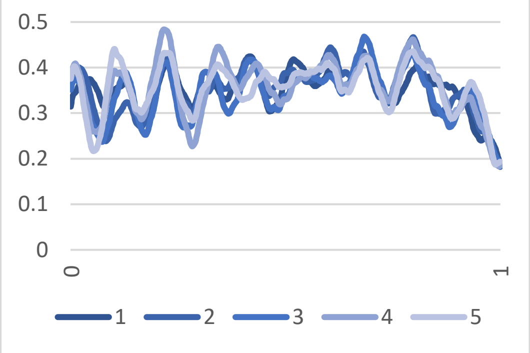	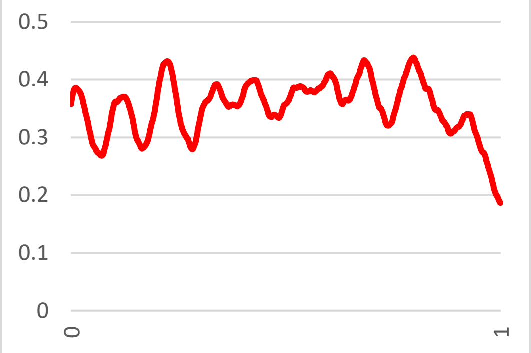
Prunus padus	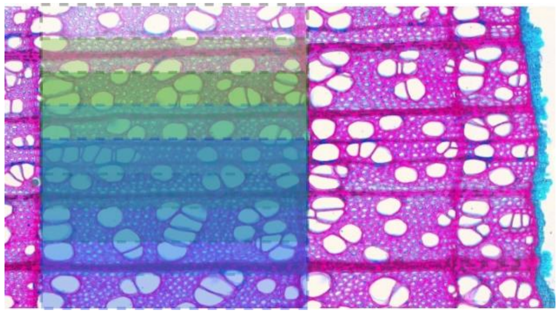	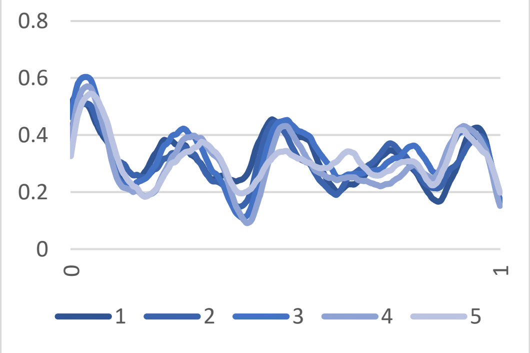	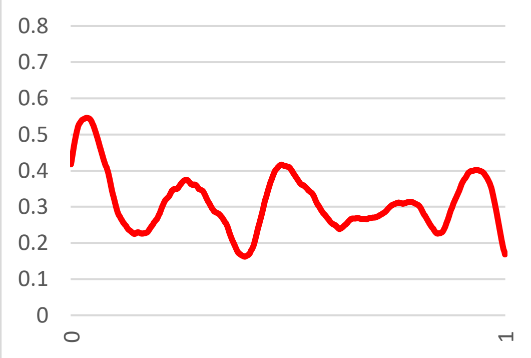

Figure S2. Continued.

Species	Photo of cell structure and individual scanning areas	Porosity measurements for five individual scanning areas (1-5)	Porosity profile averaged for five scanning areas
Rhododendron ledebourii	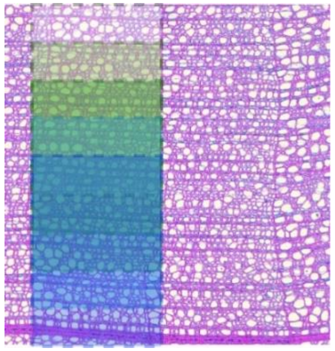	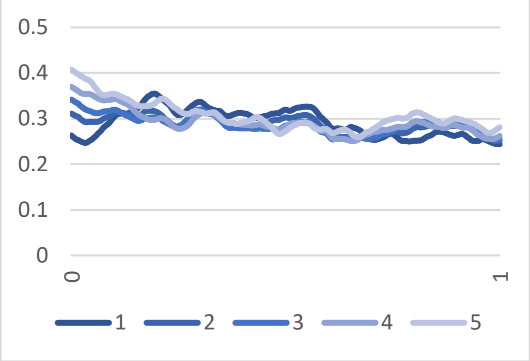	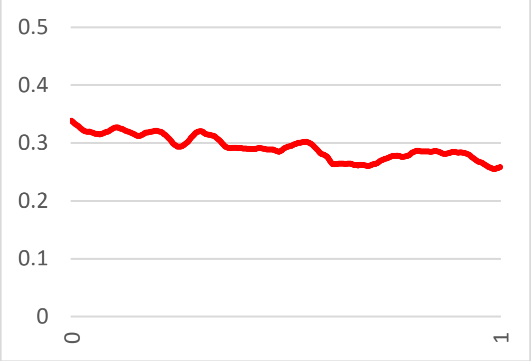
Ribes nigrum	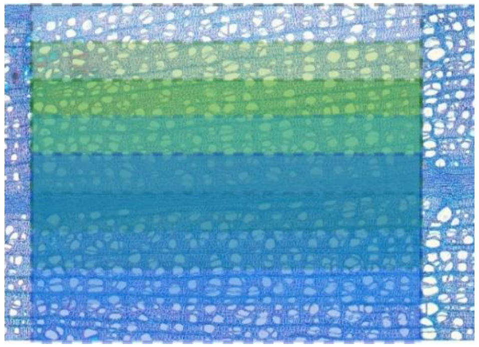	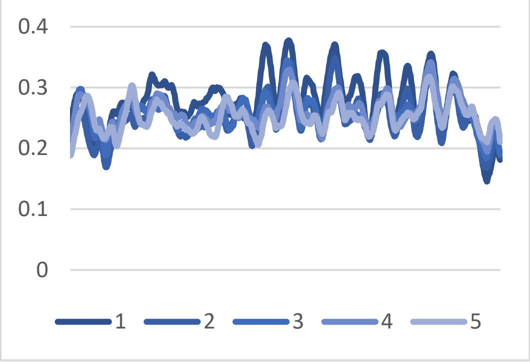	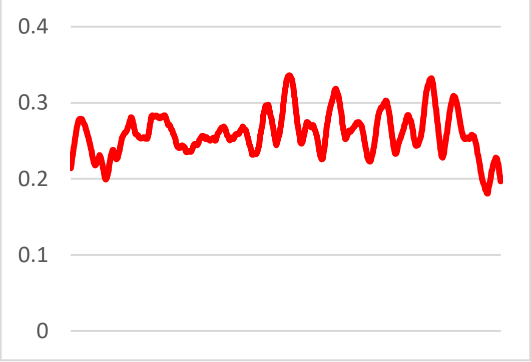

Figure S2. Continued.

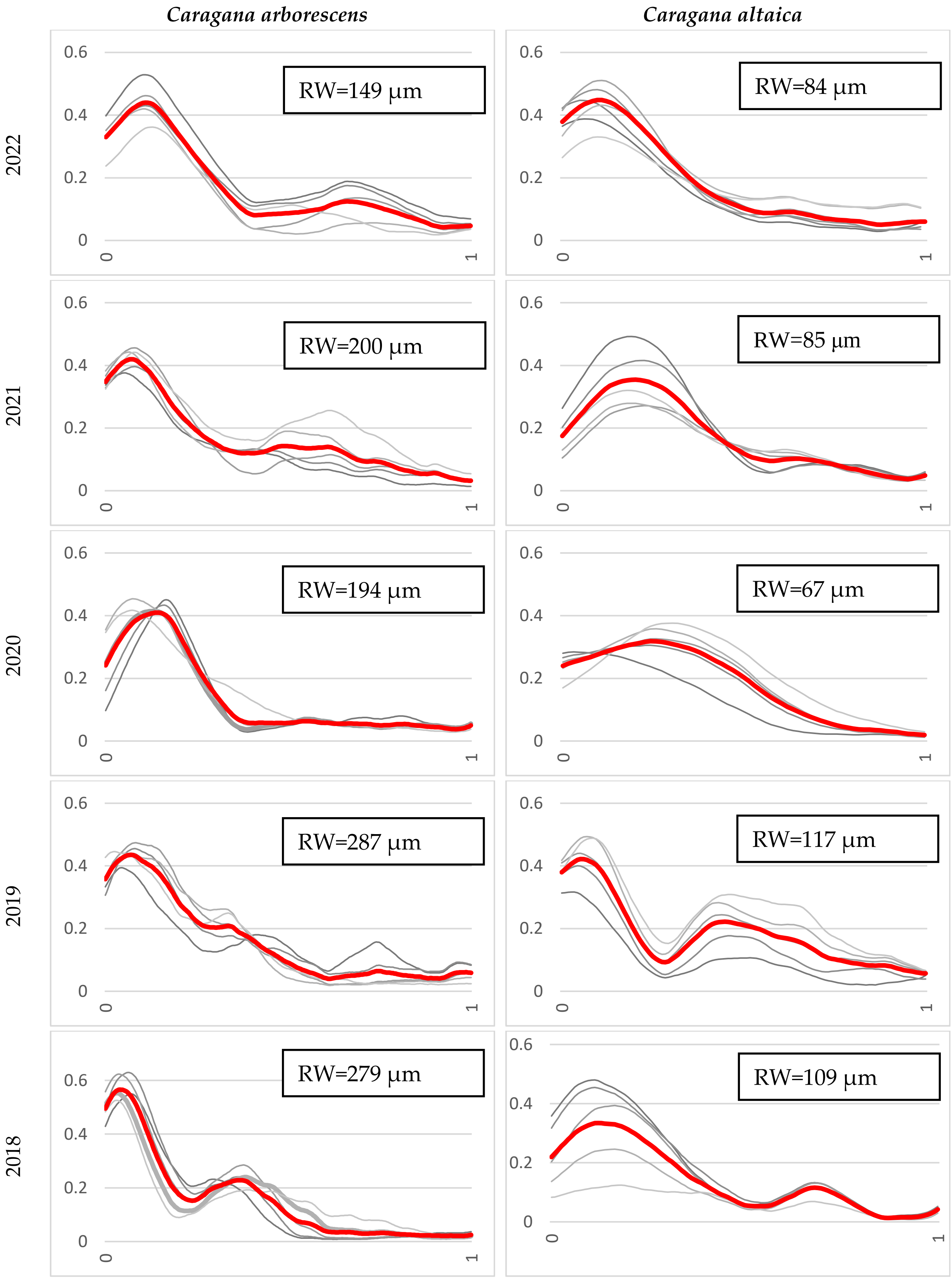

Figure S3. Wood porosity profiles of various species for 2018–2022 (individual scanning areas and averaged profile for each ring). Mean ring width (RW), calculated in micrometers from scanning area length and scale of the photograph, is represented for each ring.

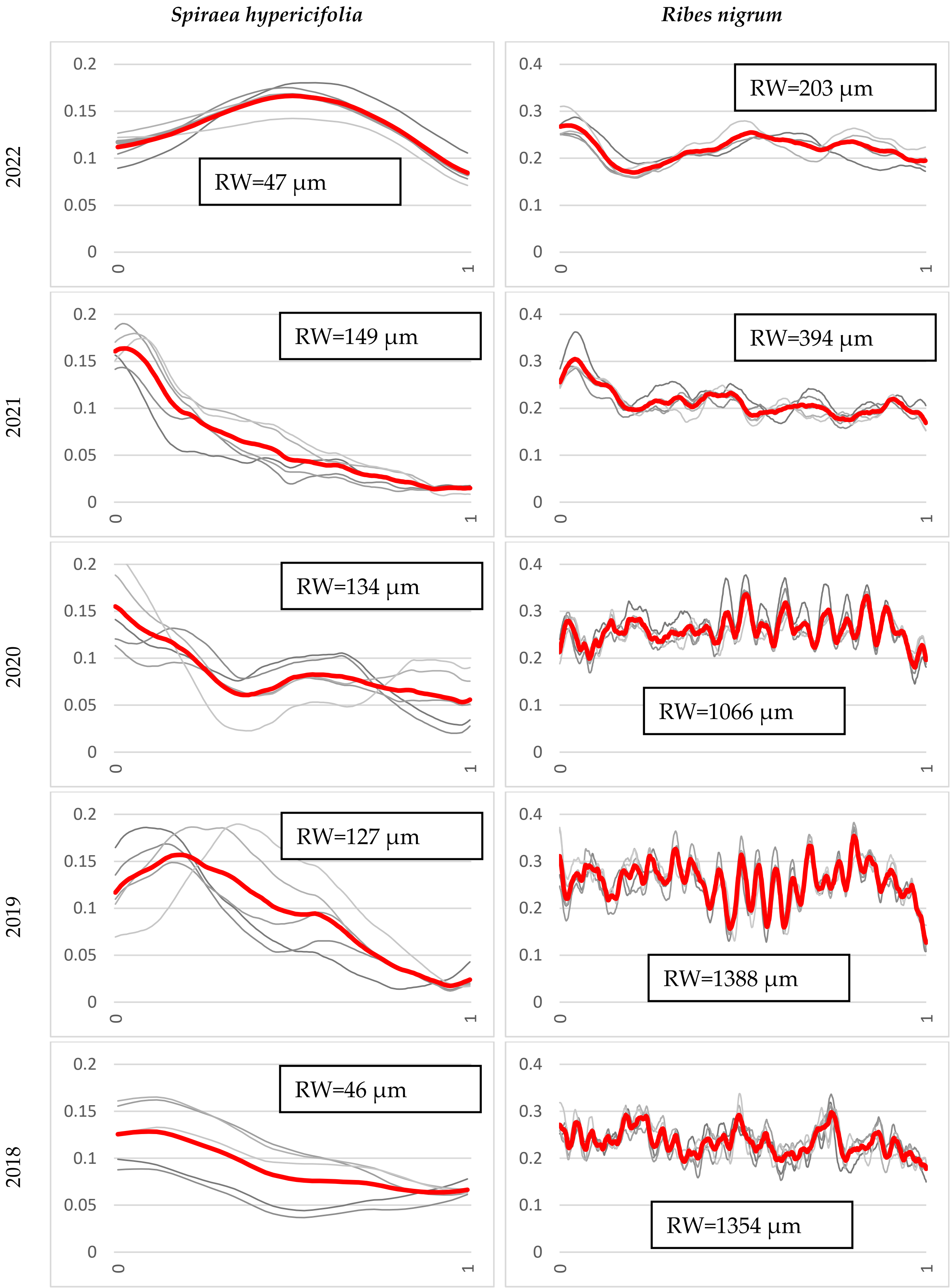

Figure S3. Continued.

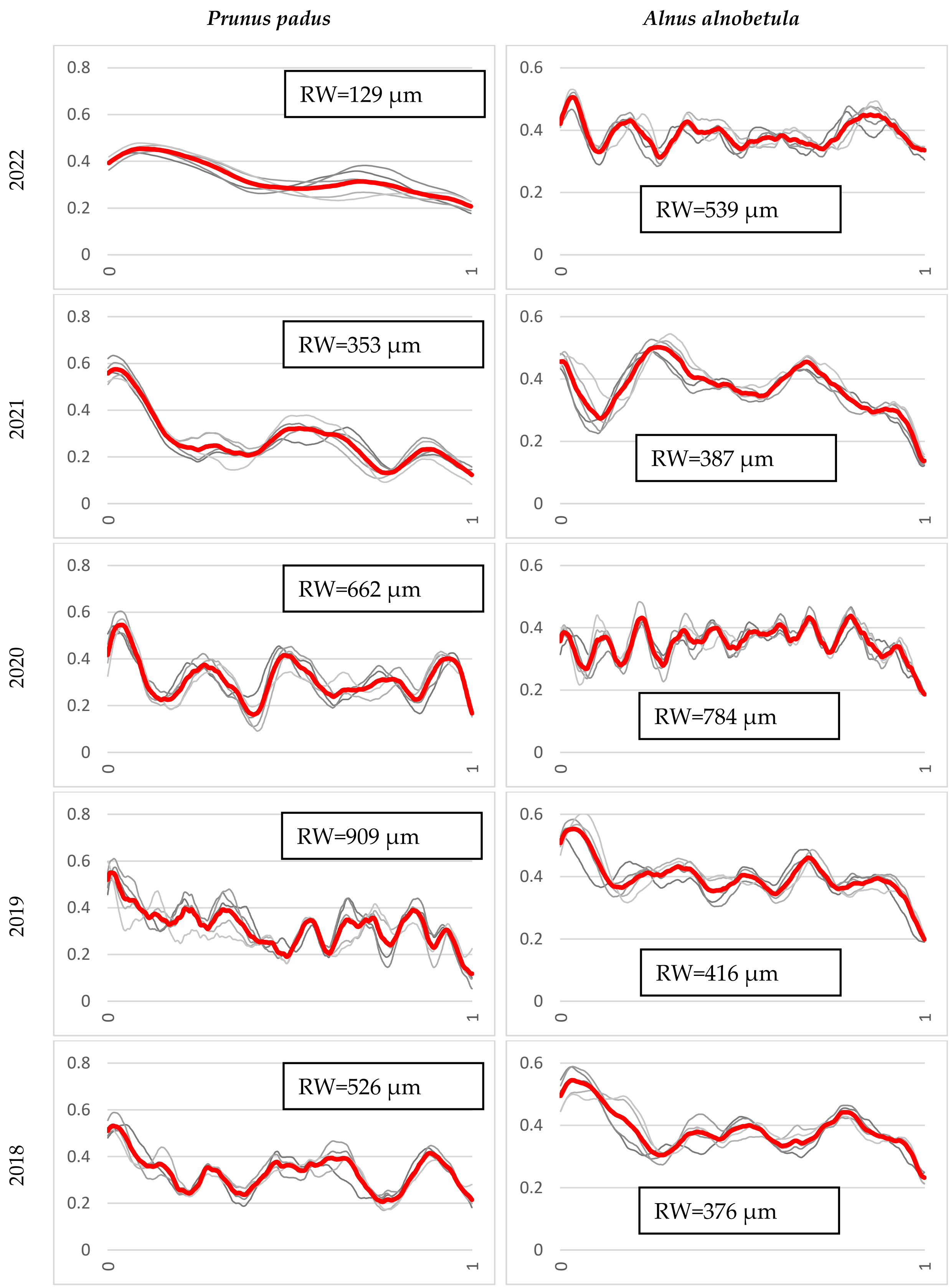

Figure S3. Continued.

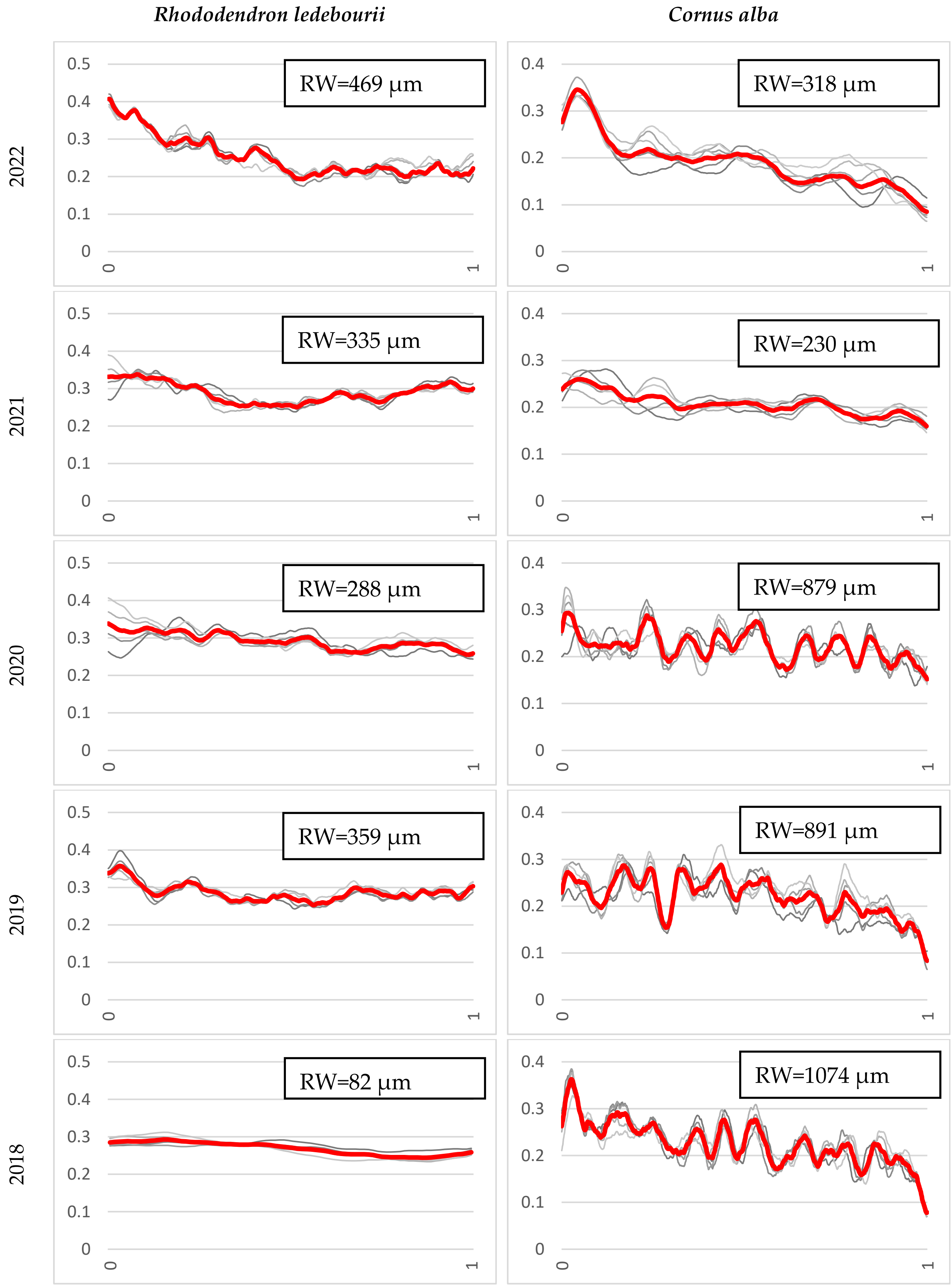

Figure S3. Continued.

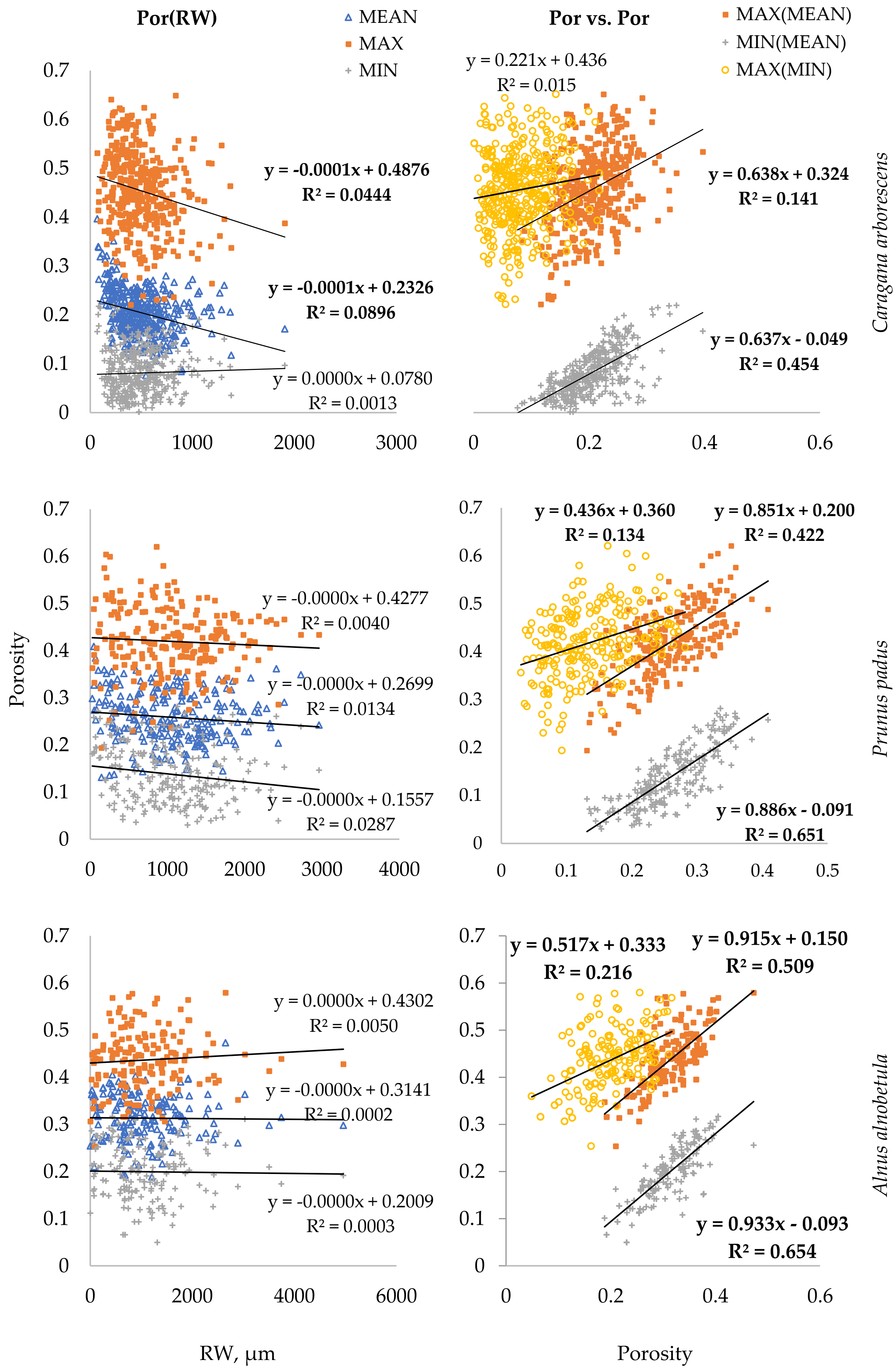

Figure S4. Dependencies of wood porosity (Por) indicators among themselves and with the growth ring width (RW). MAX, maximum porosity in the ring; MEAN, mean porosity in the ring; MIN, minimum porosity in the ring.
